# Patient characteristics of non-participants and reasons for non-participation in cardiac (tele)rehabilitation—a prospective analysis

**DOI:** 10.3389/fresc.2026.1844025

**Published:** 2026-05-22

**Authors:** Rutger F. R. van Mierlo, Fleur W. W. Heijkens-Verberkt, Vitalis J. G. Houben, Sem A. O. F. Rikken, Petros Kalendralis, Laura M. J. Hochstenbach, Jordi Lozano-Torres, Arnoud W. J. van ‘t Hof

**Affiliations:** 1 Department of Cardiology, Zuyderland Medical Center, Heerlen, Netherlands; 2 Department of Cardiology, Cardiovascular Research Institute Maastricht (CARIM), Maastricht University, Maastricht, Netherlands; 3 Department of Radiation Oncology (Maastro), Research Institute for Oncology and Reproduction (GROW), Maastricht University, Maastricht, Netherlands; 4 Department of Cardiology, Maastricht University Medical Center (MUMC+), Maastricht, Netherlands; 5 Department of Cardiology, St. Antonius Hospital, Nieuwegein, Netherlands; 6 Department of Health Services Research, Care and Public Health Research Institute (CAPHRI), Maastricht University, Maastricht, Netherlands; 7 Department of Cardiology, Hospital Universitari Vall d’Hebron, Barcelona, Spain

**Keywords:** cardiac rehabilitation, cardiac telerehabilitation, center-based cardiac rehabilitation, myocardial infaction, non-participation

## Abstract

**Introduction:**

Low participation in cardiac rehabilitation after acute myocardial infarction remains problematic. Cardiac telerehabilitation has emerged as a strategy to improve the uptake by addressing clinical, interpersonal, logistical, and healthcare system-related barriers. In this study, our aim is to determine which patient-related characteristics could lead to non-participation in cardiac rehabilitation, as well as to the reasons for non-participation.

**Methods:**

In our study, the REHAB + study, cardiac telerehabilitation was implemented in routine clinical practice. In this sub analysis, observational data were collected between November 2021 and 2024 after its implementation in Zuyderland medical center, the Netherlands. After enrolment, patients could choose to participate in center-based cardiac rehabilitation, cardiac telerehabilitation, or were non-participants.

**Results:**

A total of 328 patients with recent acute myocardial infarction were included. Two hundred (61%) patients participated in center-based cardiac rehabilitation, 64 (20%) in cardiac telerehabilitation, and 64 (20%) were non-participants. Non-participants were older (69 ± 11 years) than participants in center-based cardiac rehabilitation (64 ± 10 years) or cardiac telerehabilitation (61 ± 10 years) (*p* *<* *0.001*). There were fewer smokers in the cardiac telerehabilitation group (18%), than center-based cardiac rehabilitation (35%) or non-participation (38%). The most common reason for non-participation was a lack of interest, or a failure to acknowledge its necessity (58%).

**Discussion:**

A high proportion of our myocardial infarction population participated in either cbCR or CTR after trial enrollment. CTR offers remote-access patient education, counselling, and monitoring, which in our study was more appealing to the non-smoking population. Importantly, non-participation in cardiac rehabilitation was influenced by the aging population, mainly for lack of interest, and therefore CR programs should find ways to make their programs more appealing to the aging population. These findings support further work on patient-centered counselling and on adapting rehabilitation pathways to the needs of older patients.

## 1 Introduction

Low participation rates in cardiac rehabilitation (CR), a program with well-known health benefits (1) that improves the quality of life of patients (2), is a major problem within the field of preventive cardiovascular care. Despite evidence that shows that CR participation reduces recurrent events (2, 3), short-term (4, 5) and mid-to-long term mortality (6), adherence to CR remains poor (7–9). Participation rates to CR in the Netherlands have risen from 28% to 41% between 2013 and 2016, but have remained stagnant up until 2019 (10). Intrapersonal, sociodemographic, clinical, interpersonal, logistical, and health care system factors were all associated with non-participation amongst cardiac patients (11, 12). Cardiac telerehabilitation (CTR) may be considered to improve participation, as well as adherence to the program (1). New standards for CTR (13) could facilitate its implementation, hopefully benefiting future participation rates.

Before CTR standards were published (13), a “call to action” publication outlined strategies to implement core components of CR during the COVID-19 pandemic (14). The inability to offer CR, whether due to global, regional, or organizational factors, would be detrimental to the cardiac population. Especially since an association between the number of physical activity sessions and all-cause mortality were observed in the past (15). CTR, an effective alternative to center-based CR (16), implementation should be considered to facilitate CR uptake within a broader population.

The REHAB + trial was conducted to facilitate the coronary artery disease (CAD) population by offering a CTR program along with the standard center-based CR (cbCR) program (17). Our study closely resembles clinical practice by offering patients their own choice of CR modality, rather than randomizing them. Analyzing only the research cohort, rather than all screened participants ensures full awareness of both CR modalities and its components being offered. The goal of this study is to establish which patient-related characteristics are more likely to result in non-participation in CR, after agreeing to participate in the study. Additionally, the reasons for non-participation were collected.

## 2 Methods

### 2.1 Study design

**Figure 1:**
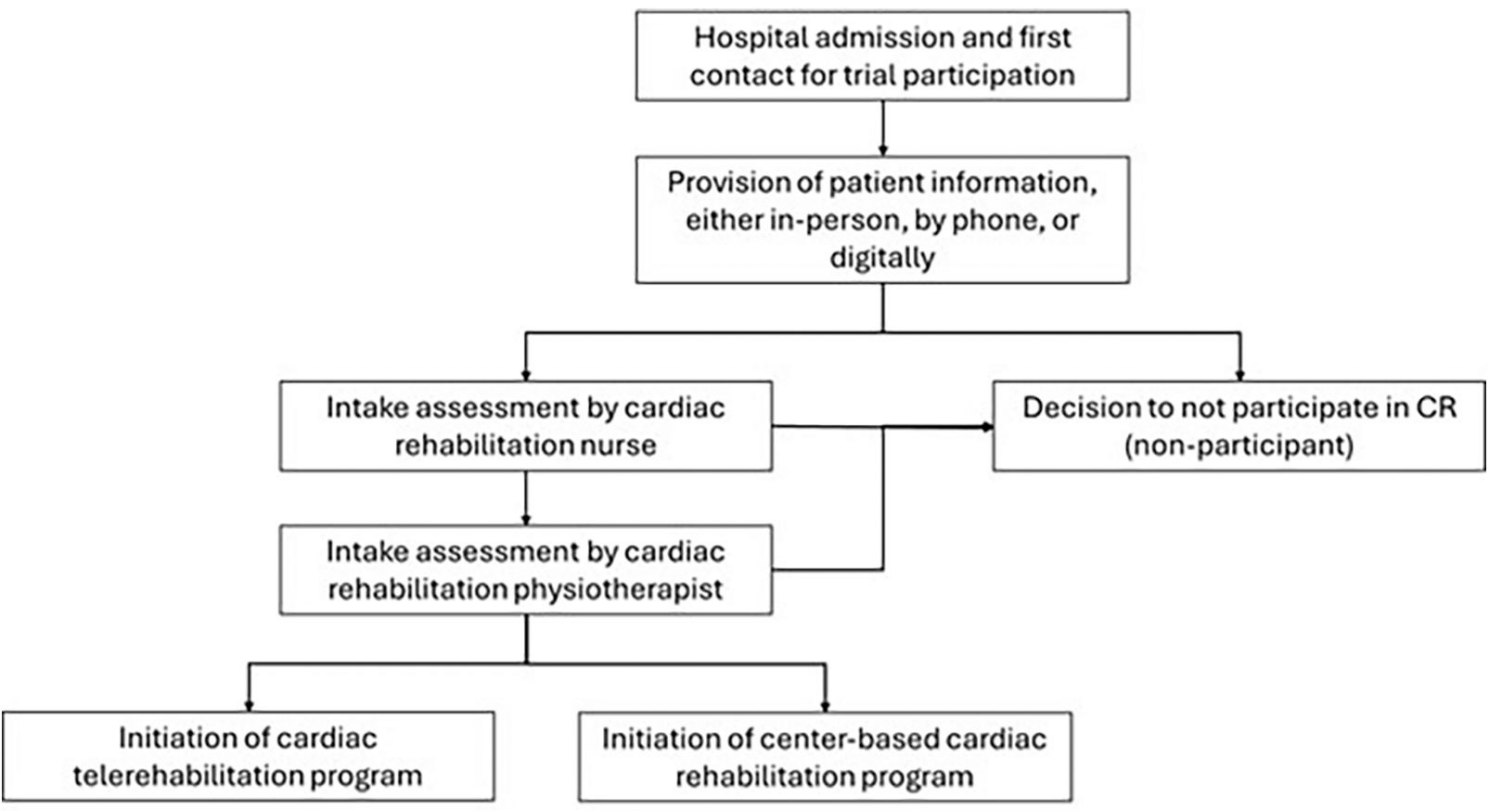
Participation flow of REHAB + in preferred CR program.

This study is a non-participation analysis from the REHAB + trial, detailed elsewhere (17). The present analysis involves observational data collected between November 2021 and February 2024 in Zuyderland medical center. Patients were screened during hospitalization, and were provided with information on both cbCR and CTR. They were provided with information on the benefits of CR, which they would not receive if deciding not to participate in any CR modality. Then, patients that provided informed consent were divided into their preferred group, which could be cbCR, CTR, or non-participation. Figure 1 shows an overview of the REHAB + study.

### 2.2 Study population

Patients hospitalized for (Non-)ST elevation myocardial infarctions were eligible for participation. Any patients with suspected safety hazards, limited prognosis, or mental impairment leading to an inability to cooperate were considered ineligible to participate in CR. The inability to exercise, understand the local language, or active participation in CR on-site or elsewhere were exclusion criteria (Table 1). Reasons for non-participation were recorded in the electronic health record.

**Table 1:** In- and exclusion criteria for cardiac (tele) rehabilitation.

Inclusion criteria	
CR	Recent myocardial infarction [(N)STEMI only]
CR	Signed written informed consent
CTR only	Smartphone capable of installing REHAB + application
Exclusion criteria	
CR	Contra-indication for CR (such as risk for safety and limited lifespan)
CR	Mental impairment leading to an inability to cooperate
CR	Severe impaired ability to exercise (including an inability to safely perform the exercise test)
CR	Insufficient knowledge of the native language
CR	Current participation in CR (at CR center or elsewhere)
CR	Participation in a CR program elsewhere after index myocardial infarction

### 2.3 Ethics statement

The study was approved by the Medisch Ethische Toetsingscommissie van Zuyderland (now Medisch Ethische Toetsingscommissie Zuyd-Máx). The study was not subject to the Medical Research Involving Human Subjects Act. The study was conducted in accordance with the local legislation and institutional requirements. The participants provided their written informed consent to participate in this study.

### 2.4 Cardiac telerehabilitation intervention

The CTR program, in line with national guidelines (18), collaborated with Liva to offer participants remote, asynchronous coaching via text and video messaging, the “REHAB + app”. Participants and coaches monitored and shared health data such as physical activity, perceived exertion (BORG scale), vital parameters (i.e., blood pressure), and lifestyle behaviors. Heart-rate monitors were provided to track physical activity; pedometers could be connected as well, if owned by the participants. Coaching input was highest in the first weeks and gradually reduced over time. Exercise programs were personalized by physiotherapists, with at least two in-person sessions focused on essential instruction and app use. Participants mainly trained at home and were able to use the REHAB + app for up to 48 weeks. Our cbCR program delivered approximately four to ten physical activity sessions of one hour per participant, depending on the training plan determined by the physiotherapist and the patient's goals. Patients would typically receive one to two sessions per week. The non-participants received educational materials before declining participation.

### 2.5 Outcomes measures

The main outcome measure was CR participation. Participants made their choice of CR program during the intake appointment with the nurse or physiotherapist. Confirmed participation in either CR program was retrospectively checked to ensure that not only intended choices were measured, but per protocol as well. We compared demographic, medical, and geographical data to determine if these characteristics were associated with allocation choice. Geographical data included distance to the nearest hospital for the patient. The hospital had two locations in which patients could attend CR. Each of these locations covered historic regions, called the “eastern mining region”, and the “western mining region”. Distance to the nearest hospital (by road) was collected for analysis.

### 2.6 Statistical analysis

A per-protocol analysis was performed. Normality was tested, and continuous variables were presented as mean (SD); categorical variables were presented as absolutes (%). Chi-squared analysis was performed on categorical variables, ANOVA on continuous variables, unless assumptions were violated, in which case their non-parametric alternatives were used.

Relevant variables were included in the multivariable model. A multinomial logistic regression analysis was performed to examine independent associations between per-protocol CR allocation and relevant variables stated in the results section. The non-participation group was considered the reference group. Model fit was assessed using likelihood ratio tests. Multicollinearity was evaluated using variance inflation factors. A significance level of *p* *<* *0.05* was considered significant for all analyses. SPSS version 29.0 was used for all analyses.

## 3 Results

**Figure 2:**
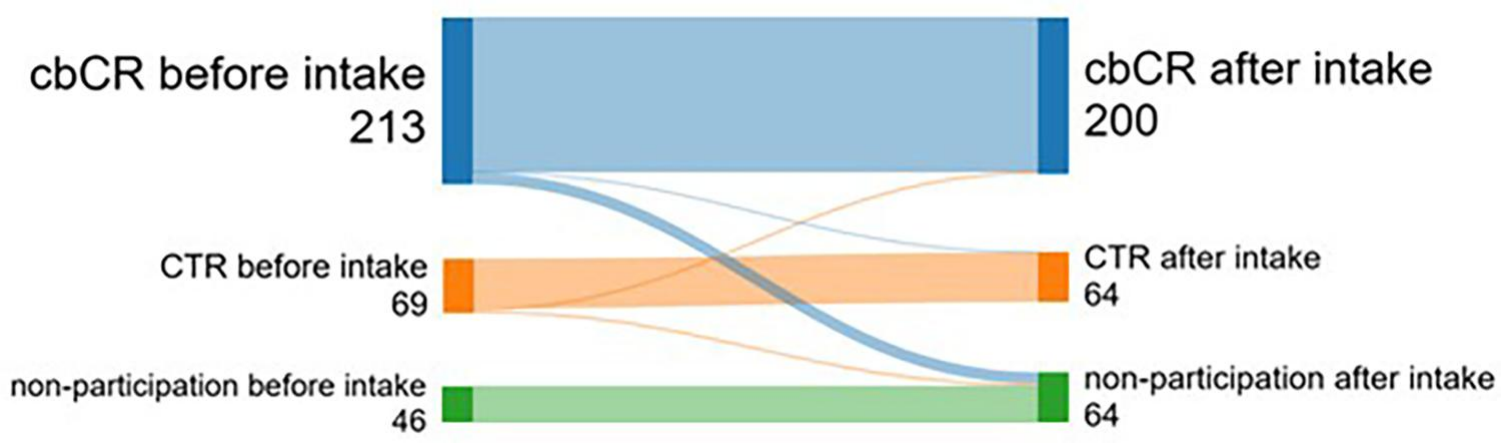
Sankey plot of REHAB + study for preferred CR allocation. Intake refers to the intake assessment by the cardiac rehabilitation nurse and physiotherapist before starting cardiac rehabilitation. cbCR, center-based cardiac rehabilitation; CTR, cardiac telerehabilitation.

In total, 328 patients provided informed consent to be part of the REHAB + study in Zuyderland MC, the Netherlands. From this cohort, 213 patients (65%) intended to participate in cbCR, 69 in CTR (21%), and 46 (14%) decided not to participate in any CR program. After the intake assessment with the CR nurse and CR physiotherapist, but before initiation of the CR program, 22 participants altered their choice. From the cbCR program, 15 participants ultimately decided not to participate in CR, and one decided to enroll in CTR. From the CTR program, three decided to enroll in cbCR, and three ultimately decided not to participate (Figure 2).

Ultimately, per-protocol, 200 patients (61%) decided to participate in the cbCR program, 64 (20%) in the CTR program, and 64 (20%) decided not to participate in any CR modality (non-participants). Most study participants were male (84%), mean aged 64 ± 10 years. Known risk factors before hospitalization were family history (57%), dyslipidemia (52%), hypertension (51%), current smoking (32%), and diabetes mellitus (16%).

Baseline characteristics per group can be found in Table 2. Before hospitalization, 71 (22%) participants were diagnosed with coronary artery disease, of whom 40 (57%) treated with PCI, 7 (10%) with coronary artery bypass grafting (CABG), 20 (19%) with both PCI and CABG, and 10 (14%) without intervention. Other relevant medical history was prevalent in less than 10% of the cohort. During hospitalization, over half (*n* = 170, 52%) of patients were diagnosed with multiple vessel disease. Most intervention strategies for the cohort were revascularization (87%), 257 (79%) patients with PCI, and 29 (9%) patients with CABG.

**Table 2:** Baseline variables of non-participation in CR.

Baseline variables	cbCR (*n* = 200)	CTR (*n* = 64)	Non-participants (*n* = 64)	*P*-value
Demographics				
Age, years (SD)	64 (10)	61 (10)	69 (11)	<0.001
Female, *n* (%)	33 (17)	6 (9)	15 (23)	NS
Employed, *n* (%)	111 (59)	38 (60)	NA	NS
Living without assistance, *n* (%)	178 (95)	61 (98)	NA	NS
Married or living together, *n* (%)	148 (78)	56 (88)	NA	NS
Distance to hospital, km (SD)	7.9 (3.5)	8.0 (4.9)	8.4 (3.8)	NS
Medical history				
Atrial fibrillation, *n* (%)	11 (6)	0 (0)	5 (8)	NS
Coronary artery disease, *n* (%)	42 (21)	9 (14)	20 (31)	NS
Congestive heart failure, *n* (%)	1 (1)	0 (0)	3 (5)	NS
Valvular disease, *n* (%)	5 (3)	1 (2)	2 (3)	NS
Prior stroke or TIA, *n* (%)	11 (6)	3 (5)	3 (5)	NS
Peripheral artery disease, *n* (%)	6 (3)	2 (3)	5 (8)	NS
COPD, *n* (%)	6 (3)	1 (2)	1 (2)	NS
Previous peptic ulcer disease, *n* (%)	3 (2)	0 (0)	1 (2)	NS
Anemia, *n* (%)	5 (3)	1 (2)	0 (0)	NS
Chronic renal insufficiency, *n* (%)	5 (3)	2 (3)	3 (5)	NS
Current malignancy, *n* (%)	5 (3)	3 (5)	3 (5)	NS
Risk factors				
Current smoker, *n* (%)	69 (35)	11 (18)	23 (38)	0.018
Hypertension, *n* (%)	101 (51)	31 (48)	36 (58)	NS
Diabetes mellitus, *n* (%)	34 (17)	6 (9)	11 (18)	NS
Dyslipidemia, *n* (%)	104 (53)	36 (56)	32 (54)	NS
Family history of CAD, *n* (%)	111 (58)	41 (65)	35 (60)	NS
Physical examination				
Weight (SD)	86 (19)	89 (15)	81 (13)	0.031
BMI, kg/height^2^ (SD)	27 (6)	28 (4)	27 (5)	NS
Systolic blood pressure, mmHg (SD)	128 (23)	127 (26)	132 (23)	NS
Diastolic blood pressure, mmHg (SD)	74 (13)	76 (13)	72 (14)	NS
CAG outcome and treatment strategy				
Elective procedure, *n* (%)	24 (12)	6 (9)	7 (11)	NS
Multiple vessel disease, *n* (%)	100 (50)	37 (58)	33 (53)	NS
Left main, *n* (%)	15 (8)	2 (3)	5 (8)	NS
Revascularization, *n* (%)	172 (86)	61 (95)	52 (81)	NS
STEMI, *n* (%)	86 (43)	29 (45)	30 (47)	NS
Echocardiogram				
Left ventricular ejection fraction, % (SD)	50 (7)	51 (5)	49 (7)	NS
Laboratory tests				
Total cholesterol in mmol/L	4.7 (1.1)	4.6 (1.2)	4.6 (1.2)	NS
High-density lipoprotein in mmol/L	1.1 (0.3)	1.1 (0.3)	1.1 (0.3)	NS
Low-density lipoprotein in mmol/L	2.9 (1.0)	2.8 (1.1)	2.7 (1.1)	NS
Triglycerides in mmol/L	1.5 (0.8)	1.6 (1.0)	1.8 (0.9)	0.037
HbA1C in mmol/L	46 (14)	41 (9)	46 (15)	NS
eGFR in ml/minute/1.72m^2	78 (15)	77 (16)	70 (17)	0.001

Non-participants were significantly older (69 ± 11 years) than cbCR participants (64 ± 10 years) or CTR participants (61 ± 10 years) (*p* *<* 0.001). There were fewer current active smokers in the CTR group (18%), compared to the cbCR group (35%), or the non-participants (38%) (*p* *=* 0.018). Non-participants presented with lower body weight (81 ± 13 kilogram) compared to the cbCR group (86 ± 19 kg) and the CTR group (89 ± 15 kg) (*p* *=* 0.031), however no significant differences were found in body mass index. Significantly higher triglycerides (1.8 ± 0.9 mmol/L) and eGFR (70 ± 17 mmol/minute/1.72m^2^) were observed in the non-participation group. No significant differences were found between groups in other demographics or risk factors, as well as distance from their residence to the hospital (Table 2).

Relevant variables were chosen, either being known predictors from previous research, or suspected by clinical relevance and prevalence in this cohort. The variables included for the multinomial logistic regression analysis were age, sex, distance to the hospital, history of coronary artery disease, hypertension, dyslipidemia, diabetes mellitus, current smoking, family history, body mass index, one-vessel vs. multivessel disease, and revascularization vs. non-revascularization. The overall model was significant (df = 24, *p* *<* 0.001) and explained a low 18% of the variance (Nagelkerke R^2^ = 0.182). The likelihood ratio tests indicated that age (*p* *<* 0.001) and current smoking (*p* *<* 0.001) were significantly associated with non-participation. Only age was associated with higher odds for non-participation compared to cbCR (*p* *=* 0.018, 95% CI 0.922; 0.992), whilst both age (*p* *<* 0.001, 95% CI 0.864; 0.950) and current smoking (*p* *<* 0.001, 95% CI 0.052;0.382) were associated compared to CTR (Table 3).

**Table 3:** Multinomial logistic regression analysis of non-participation in CR.

Covariates	cbCR			CTR		
	Estimate	SE	95% CI	Estimate	SE	95% CI
Intercept	3.962	1.727		3.896	2.263	
Current age (years)	−0.044	0.019	0.922; 0.992	−0.098	0.024	0.864; 0.950
Sex	0.338	0.396	0.645; 3,046	1.008	0.578	0.883; 8.501
Distance to the hospital (kilometers)	−0.026	0.042	0.898; 1.058	0.006	0.052	0.908; 1.115
History of coronary artery disease	−0.451	0.381	0.302; 1.343	−0.606	0.532	0.192; 1.549
Current smoking	−0.496	0.354	0.304; 1,220	−1.965	0.511	0.052; 0.382
History of hypertension	−0.162	0.362	0.418; 1.730	−0.247	0.458	0.319; 1.916
History of diabetes mellitus	−0.066	0.425	0.407; 2.155	−0.925	0.602	0.122; 1.290
History of dyslipidemia	0.441	0.363	0.763; 3.163	0.759	0.458	0.871; 5.237
Family history for cardiovascular disease	−0.177	0.331	0.438; 1.602	0.054	0.421	0.462; 2.411
Body mass index (kilograms/m^2)	0.006	0.030	0.948; 1.067	0.025	0.040	0.948; 1.110
Single vessel vs. multivessel disease	−0.070	0.337	0.482; 1.806	0.383	0.425	0.638; 3.372
Revascularization	0.307	0.433	0.582; 3.175	1.351	0.729	0.925; 16.112

Non-participants were the reference category. cbCR, center-based cardiac rehabilitation; CTR, cardiac telerehabilitation; SE, standard error.

**Figure 3:**
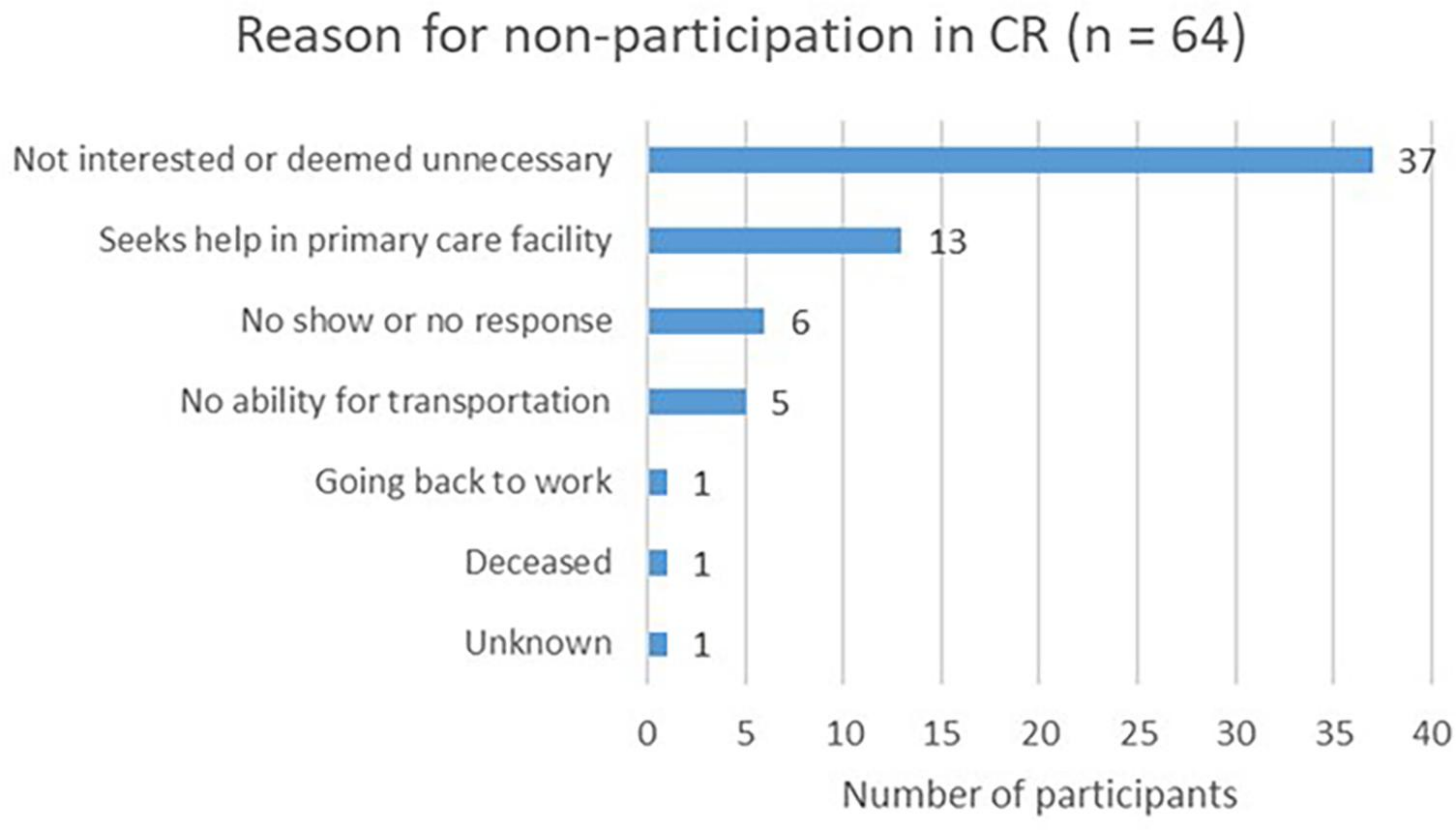
Reported reasons for non-participation in CR.

The most common reported reason for non-participation in CR was that patients were uninterested, or deemed CR unnecessary (*n* = 37, 58%). A smaller proportion of the non-participants sought help in a primary care facility, such as an independent physiotherapist (*n* = 13, 20%). A few participants were no-shows or did not respond when the CR nurses reached out to them for initiating the CR program (*n* = 6, 9%). Another few were unable to facilitate transportation to the CR (*n* = 5, 8%). One participant went back to work, one passed away, and one participant's reason remained unknown (Figure 3).

## 4 Discussion

Our study collected patient-related characteristics and reasons for non-participation at the time of enrolment in the REHAB + trial; details of the full trial design can be found elsewhere (17). We found that over 80% of our study participants, having knowledge on both CR options before choosing, chose to enroll in either cbCR or CTR. Older patients and patients with a lower body weight were more likely to be non-participants, and active smokers were less likely to participate in the CTR program. This is in line with previous research (19). Higher triglycerides and lower eGFR in the non-participation group were likely attributable to older age. Although non-participants had lower body weight, their body mass index was comparable to cbCR and CTR participants, which would suggest their bodies being frailer or sarcopenic. Most non-participants were uninterested in CR or deemed CR unnecessary. There were few participants that reported logistical reasons for non-participation. The implementation of CTR might have taken away some of the logistical problems. However, we could not attribute this to the distance patients lived from the hospital.

In line with other research in CTR (20), or CR as a whole (21), we found that the older population were less likely to participate. The analysis by Brouwers et al. (19) found a more extensive patient profile that was associated with non-participation. They found that non-highly educated, non-smoking patients, with no family history of CVD, and no previously diagnosed hypertension, who underwent PCI, were less likely to participate in their Smart-Care-CAD trial. This could be considered a surrogate to participate in cardiac telerehabilitation. Relevant differences in our trial were that participants were able to choose CR modality, rather than being randomized. This closely resembles daily practice and might have influenced participation in our study. Moreover, our cohort size was approximately half the size of Brouwers et al., which could have concealed true differences between groups that might be prevalent in the total population. Nevertheless, an effort should be made to facilitate CR programs for the aging population.

The burden on healthcare systems will likely continue to rise due to the ageing global population, even with mortality rates improving when adjusting for age (22). Both CR modalities have been shown to be beneficial to the older population i.e., in terms of functional capacity (23) or all-cause mortality (24). In fact, a recent randomized controlled trial by Snoek et al. (23), showed that a CTR program with telemonitoring and coaching through motivational interviewing, compared to no CR, effectively improved physical fitness through VO_2_peak testing after six months in the elderly. A meta-analysis of digital health interventions for CR showed similar results, in addition to improvements in the 6-minute walk test and health related quality of life (25). Another systematic review and network meta-analysis reported improvements in VO2peak, 6 min walking distance, moderate-to-vigorous intensity physical activity, and adherence as well (26). Therefore, we believe an effort should be made by providers of CR to facilitate a dedicated CTR program, as a moderate proportion of our eligible patients made the conscious decision to enroll in CTR.

We found a significantly lower proportion of smokers in our CTR program compared with both our center-based CR program and non-participation. As smoking prevalence was similar between center-based CR participants and non-participants, this finding less likely supports the notion that smokers are less motivated to engage in CR overall, even though this was reflected in previous research (27). A possible explanation is that CTR is more appealing to individuals with greater interest in digital health or novel care models, which may be less common among smokers.

It remains important to consider how CR programs are structured in clinical practice. The latest standards for cardiac telerehabilitation (13) were made available to propose minimal and optimal requirements for CTR to combat its low rate of implementation. CTR programs are recommended to incorporate the core components of cardiac rehabilitation, including cardiovascular risk factor control, guideline-directed medical therapy, patient assessment, psychosocial support, exercise prescription and counseling, smoking cessation, patient education, and vocational reintegration. Our CTR program complied with the minimal core components, which initiated with a comprehensive, on-site evaluation with exercise testing, as well as questionnaires on quality of life, physical activity, and nicotine dependency. Physical activity counselling included exercise tracking using a heart rate monitor, and counselling occurred asynchronous or on-site. The first exercise training sessions occurred on-site, before transitioning to remote training. Nutritional, weight loss, psychosocial, and/or smoking cessation counseling were provided through a digital library of educational information, or through a referral, to the nutritionist, smoking cessation interventionist, or psychologist. Moreover, vocational reintegration was not directly embedded within the digital intervention, and patients were redirected to the occupational health doctor, if necessary. Medication education for lipid management or blood pressure measurement occurred in the first session with the CR nurse, whilst medication up titration and follow-up were monitored at the outpatient clinic.

In a recent systematic review and meta-analysis (28), treatment dose and duration varied between trials. Our cbCR program typically lasted for four to six weeks (usually with four to ten physical activity training sessions). Patients were able to participate in our CTR program for 48 weeks. A dose-response association was demonstrated between a high number of cbCR session attendance and fewer major adverse cardiovascular events, so far without ceiling effect (29). However, it is unknown if this dose-response effect translates to CTR sessions, which typically offer more sessions than their cbCR counterparts. We believe that through shared decision making and through a multidisciplinary team meeting the mode or CR should be chosen.

Finally, it is very hard to prove that offering CTR leads to increased participation rates in CR. Nevertheless, 20% of all patients participated in our CTR program. It remains unclear whether these patients would participate in cbCR if CTR would not be an option. Previous research has found that non-referral is a substantial factor in non-participation (20). In this study, all hospitalized patients on the wards were screened by the cardiac rehabilitation nurses for eligibility, likely contributing to the high rate of participation in our cohort. Nevertheless, It remains hard to motivate older patients to participate in CR. Offering CTR does not seem to improve participation rates for this subgroup, although previous research by Snoek et al. has shown elderly patients can successfully participate in CTR (23). We hope our study contributes to the implementation of CTR by closely resembling daily practice. Our study complies with Dutch and European guidelines (30), limiting heterogeneity. Still, our prospective study is in line with previous findings found in randomized controlled trials about non-participation in cardiac (tele)rehabilitation (31). This strongly affirms the need for implementation studies involving underrepresented groups in designing healthcare pathways for cardiac (tele)rehabilitation.

### 4.1 Limitations

The nature of our research introduces the likelihood of the Hawthorne effect occurring, even after explicit instructions to avoid biased communication towards participants. Participation in any form or CR was encouraged, however. Self-selection bias in our cohort of participants will likely have inflated the CR participation rate, although the amplitude of its effect is impossible to objectify without data to support it. An analysis of the total cohort of acute myocardial infarction patients during the time of inclusion could be interesting to analyze. However, this type of analysis would include patients that might be deemed not eligible to participate in CR, which would falsely underestimate CR participation rate. Additionally, we could have bolstered our data by allowing CR participants to elaborate on their motivation for their choice of CR modality, especially as to why patients decided to enroll in the CTR program. Understanding our patients’ perspective and motivation on their choice of CR program could benefit future improvements to the information provided prior to enrollment. Lastly, patients with lower socioeconomic status have been shown to be less likely to be referred to, enroll in, or participate in cardiac rehabilitation (32). Collecting this type of data in the future should provide a more thorough understanding of non-participation in CR, especially as the “eastern mining region” and “western mining region” in the Netherlands varies in socio-economic status.

## 5 Conclusion

A high proportion of our myocardial infarction population participated in either cbCR or CTR after trial enrollment. CTR offers remote-access patient education, counselling, and monitoring, which in our study was more appealing to the non-smoking population. Importantly, non-participation in cardiac rehabilitation was influenced by the aging population, mainly for lack of interest, and therefore CR programs should find ways to make their programs more appealing to the aging population. These findings support further work on patient-centered counselling and on adapting rehabilitation pathways to the needs of older patients.

## Data Availability

The raw data supporting the conclusions of this article will be made available by the authors, without undue reservation.
